# Vitamin C in the Treatment of COVID-19

**DOI:** 10.3390/nu13041172

**Published:** 2021-04-01

**Authors:** Gregorio Paolo Milani, Marina Macchi, Anat Guz-Mark

**Affiliations:** 1 Pediatric Unit, Fondazione IRCCS Ca’ Granda Ospedale Maggiore Policlinico, 20122 Milan, Italy; milani.gregoriop@gmail.com (G.P.M.); marina.macchi@studenti.unimi.it (M.M.); 2 Department of Clinical Sciences and Community Health, Università degli Studi di Milano, 20122 Milan, Italy; 3 Institute of Gastroenterology, Nutrition and Liver Diseases, Schneider Children’s Medical Center of Israel, Petach-Tikva 4920227, Israel; 4 Sackler Faculty of Medicine, Tel-Aviv University, Tel-Aviv 6997801, Israel

**Keywords:** ascorbic acid, SARS-CoV-2, antioxidant, immune regulation

## Abstract

Vitamin C is an essential nutrient that serves as antioxidant and plays a major role as co-factor and modulator of various pathways of the immune system. Its therapeutic effect during infections has been a matter of debate, with conflicting results in studies of respiratory infections and in critically ill patients. This comprehensive review aimed to summarize the current evidence regarding the use of vitamin C in the prevention or treatment of patients with SARS-CoV2 infection, based on available publications between January 2020 and February 2021. Overall, 21 publications were included in this review, consisting of case-reports and case-series, observational studies, and some clinical trials. In many of the publications, data were incomplete, and in most clinical trials the results are still pending. No studies regarding prevention of COVID-19 with vitamin C supplementation were found. Although some clinical observations reported improved medical condition of patients with COVID-19 treated with vitamin C, available data from controlled studies are scarce and inconclusive. Based on the theoretical background presented in this article, and some preliminary encouraging studies, the role of vitamin C in the treatment of patients with SARS-CoV-2 infection should be further investigated.

## 1. Introduction

Vitamin C (ascorbic acid) is a water-soluble vitamin that plays a major role as antioxidant and as co-factor of various biosynthetic pathways in the immune system. It is an essential nutrient that cannot be synthesized by the human body [1]. Its antioxidant effect is derived by the ability to donate electrons and thus protect molecules from oxidative damage. Besides its importance in maintaining epithelial barrier of the skin, vitamin C has major roles in the function and regulation of the immune system. Leukocytes and neutrophils accumulate vitamin C intracellularly, dependent on its plasma availability. In neutrophils, vitamin C influences the chemotaxis process as well as phagocytosis of microbes. In addition, due to its antioxidant and scavenging ability, vitamin C protects neutrophils and phagocytes from the damage that occurs after their oxidative burst, and also activates a caspase-dependent cascade that promotes programmed apoptosis and inhibits necrosis [2,3]. Similar effect of protection from oxidative stress is also observed in lymphocytes. Other influences of vitamin C on inflammatory regulation involve modulation of nuclear transcription factor kappa B (NFkB) and attenuation of pro-inflammatory cytokines production [4].

Vitamin C deficiency and its clinical syndrome scurvy are associated with susceptibility to infections, in particular respiratory tract infection and pneumonia. During infection, the antioxidant role of vitamin C may be most prominent as the oxidative stress is elevated [5]. The enhanced requirement of antioxidants and consumption by leukocytes could explain the reduction in vitamin C levels observed during infections in general, during lung infections in particular [6,7], and in critically ill patients [8,9]. Beyond the antioxidative effect, the beneficial functions of vitamin C during pneumonia are found to act via signaling pathways of inflammation suppression and enhancement of immunoregulation [10].

The use of vitamin C supplementation to prevent or shorten the duration of respiratory tract infections and the common cold have been a matter of debate for decades, with conflicting results in different studies [11,12]. A comprehensive Cochrane review concluded that some reduction in duration and severity of common cold can be observed in regular supplementation trials, without consistent effect in therapeutic trials [13]. People under extreme physical stress may merit special consideration regarding the benefit of vitamin C supplementation. As for pneumonia, data regarding oral supplementation are scarce and based mostly on small studies and observations. A recent Cochrane review and meta-analysis on this topic concluded that the current evidence is insufficient to support beneficial effect of vitamin C supplementation on prevention and treatment of pneumonia, and larger high-quality studies are needed [14].

As opposed to the mild and controversial effects of oral vitamin C supplementation, an intravenous administration of high doses of vitamin C may lead to higher plasma levels by bypassing the limits of intestinal transporters. Several trials of high-dose intravenous administration of vitamin C have shown mixed results regarding laboratory and clinical outcomes in different settings, including patient with severe sepsis in intensive care units (ICU), patients with acute lung injury, and acute respiratory distress syndrome (ARDS) [15,16,17]. A systematic review and meta-analysis on intravenous vitamin C treatment in critically ill patients, published in 2017, has demonstrated favorable results in vasopressor sparing and in a reduced need for mechanical ventilation, without affecting overall mortality [18]. A more recent systematic review and meta-analysis showed no significant effect of intravenous vitamin C treatment in critically ill patients in terms of infection episodes, ICU or hospital length of stay and duration of mechanical ventilation, but a mild tendency towards mortality reduction [19]. On the contrary, a reduction in the duration of mechanical ventilation was found in a different recent meta-analysis [20].

Based on this theoretical basis and previous experience using vitamin C in critically ill patients and in patients with respiratory infections, and facing the huge burden of morbidity and mortality secondary to CoronaVirus Disease 19 (COVID-19) pandemic worldwide, a large interest is present in the possible beneficial role of treatment with vitamin C in the setting of Severe Acute Respiratory Syndrome Coronavirus 2 (SARS-CoV2) infection. Over the past year several publications emerged, reporting treatment with vitamin C in patients with COVID-19 in different settings. Most publications reported single cases or small case-series; some conducted observational studies, while clinical trials are scarce are many ongoing trials still pending results. We hereby present a comprehensive review summarizing the available data in the literature of the experience with vitamin C treatment in patients with SARS-CoV2 infection.

## 2. Material and Methods

In this comprehensive literature review two electronic databases (PubMed and EMBASE) were searched from 1 January 2020 to 15 February 2021 using the following search strategy: (“ascorbic acid” OR “vitamin C” OR “vitamin-C” OR “vit C” OR “L-ascorbic”) AND (“coronavirus” OR “corona virus” OR “COVID” OR “COVID19” OR “COVID 19” OR “COVID-19” OR “SARS-CoV2”). Two authors (MM and AGM) performed the search and independently screened all studies identified in the databases. In cases of discrepancies in study identification, all eligible studies were included after the authors’ revision.

**Figure 1:**
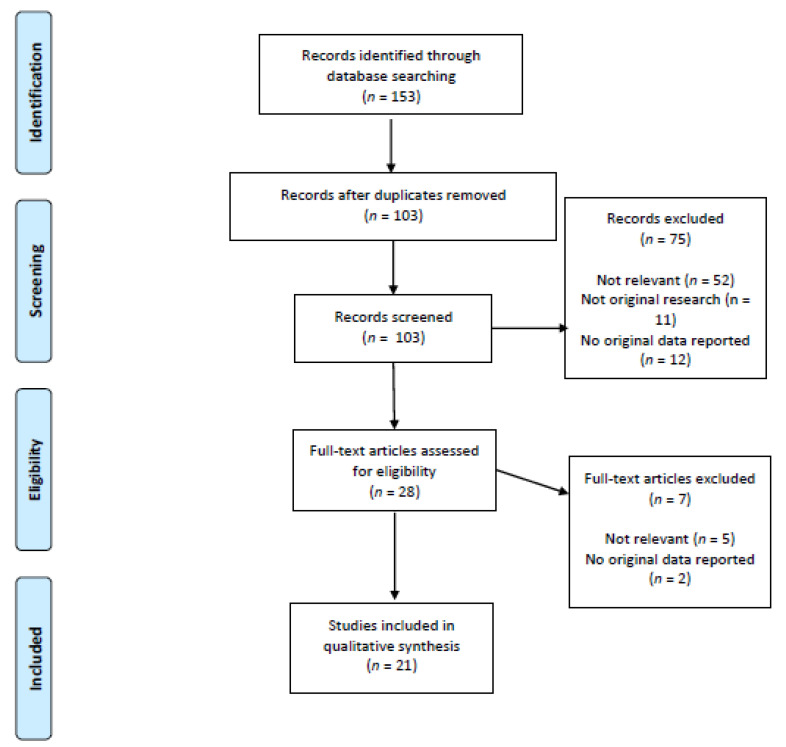
PRISMA flowchart.

All types of study were included if they were published in English, the study population consisted of human patients (children or adults) with SARS-CoV-2 infection, and vitamin C was used as either a therapeutic or prophylactic intervention. From each included study, the following information was extracted: title, author name and year of publication, country of the study, patients’ demographics and baseline clinical conditions, vitamin C dose and duration of therapy, outcomes, and conclusions. Study selection is outline in a PRISMA flow-chart in Figure 1.

## 3. Results

The initial study search resulted in 153 studies. After reading the titles and abstracts and removing duplicates (*n* = 50), the selection process led to the inclusion of 21 studies. Six were case-report, three were case-series, three were observational retrospective studies, one was a cross-sectional study, one was a phase-1 clinical trial, four were randomized clinical trials (RCTs), and three were ongoing RCTs. While the majority of the studies focused on the therapeutic effects of vitamin C, only one article reported its effects on prevention of COVID-19. The countries in which the included studies were performed were the USA (*n* = 8) and China (*n* = 7), followed by Iran (*n* = 2), Pakistan (*n* = 2), Italy (*n* = 1), and Tunisia (*n* = 1). In all of the aforementioned studies and reports, the total number of patients with SARS-CoV-2 infection who received vitamin C was 568. In 14 studies the patients were in critical condition, and death occurred in 9 patients. However, for some studies incomplete data were available. No studies were found reporting the use of vitamin C for prevention of COVID-19.

### 3.1. Case Reports and Case Series

Several single- and multiple-case publications reported an improved clinical condition after vitamin C treatment, mainly in reduced duration of mechanical ventilation and hospital stay or earlier recovery of symptoms.

In one case report, a 74-year-old woman from the USA with COVID-19 ARDS received vitamin C first as an oral 1 g twice/day dose (for 6 days), and later as a high intravenous (IV) dose (dose: 11 g/day as a continuous infusion, for 10 days) due to the development of ARDS and septic shock [21]. Within 5 days the patient’s clinical status improved and she was able to wean mechanical ventilation.

A case series from the USA reported administration of IV vitamin C (dose: 1 g every 8 h, for 3 days) to 17 patients who tested positive for SARS-CoV-2 (mean age 64 ± 14 years; M:F = 10:7) [22]. The treatment with vitamin C was started at a median of 3 days (range 0–11) after hospital admission and 8 days (range 3–18) after symptom onset. The authors reported a significant decrease in ferritin and D-dimer levels and a trend of decreasing fraction of inspires oxygen (FiO_2_) requirements after vitamin C administration.

A single case-report from China reported a previously healthy young (34 years old) male patient with no comorbidities with COVID-19-associated symptoms (dry cough, fatigue, poor appetite and subjective fever) who received vitamin C (dose: 3 g once daily) together with antiviral and antimicrobial treatments [23]. After two weeks of combined treatment, the patient’s symptoms and SpO_2_ improved and he was considered clinically cured.

In another single case report from China, vitamin C (dose: 200 mg for 3 times/day, orally) was administered with oral diammonium glycyrrhizinate (a Chinese traditional steroid-like molecule) in a female patient (66 years old) with severe COVID-19–related shortness of breath and persistent fever [24]. Her history was particular because she never had positive PCR test results, but her disease was confirmed by antibody test post-recovery. In a week all her severe symptoms were resolved, and she fully recovered.

In a Chinese retrospective case series of 12 patients with severe (*n* = 6, median age 56 years old (IQR 32–65)) or critical (n = 6; median age 63 years old (IQR 60–82)) COVID-19 pneumonia [25], high dose IV vitamin C was administered within 24 h after disease aggravation. The median interval from first symptom to vitamin C initiation was 3.5–8 days in the severe group and 5–19 days in the critical one while the median dosage of vitamin C was respectively 162.7 mg/kg/day (71.1–328.6) and 178.6 mg/kg/day (133.3–350.6). The administration of vitamin C was associated with a significant decrease in C-reactive protein, lymphocyte and CD4+ T cell counts, as well as an improvement of PaO_2_/FiO_2_ and organ failure assessment score (SOFA), which was better in the severe group compared to critical one.

In addition, the use of vitamin C (dose: 3 g/day) in two patients with SARS-CoV-2 infection and pulmonary capillary leak syndrome and who had a good outcome was described in a case series study from Tunisia [26]. On the other hand, some other studies did not find any positive outcome associated with vitamin C administration. Two different case reports from the USA reported mortality in critically ill patients who were also treated with vitamin C. In one case-report [27], a male patient of 29 years of age with COVID-19 developed pneumonia complicated by severe ARDS and multi-organ failure. He received vitamin C treatment together with inhalation therapy and supportive measures, but his status worsened despite these therapies and he died. Similarly, in another case-report, a 77-year-old female received high dose IV vitamin C (6 g for 2 times/day, then 1 g/day) together with antiviral and other treatments [28]. The patient developed organ failure and died despite these efforts.

An adverse effect associated with high-dose intravenous vitamin C administration (dose: 200 mg/kg/day, for 96 h) was reported in an Italian study on two patients with COVID-19-related sepsis [29]. The patients developed an acute tubular injury (ATI) and oxalate nephropathy after 2 and 8 days of treatment. The first patient was a 50 years old man who received a high dose vitamin C (total dose: 112 g) which was unsuccessful to prevent septic shock. He developed an acute kidney injury, but the respiratory status improved and was later extubated and discharged home. The other patient was a 71-year-old man (total dose: 160 g) who developed a renal failure needing kidney replacement through hemodialysis. However, the patient’s respiratory status improved and he was discharged home. Both patients’ kidney biopsy specimens showed extensive ATI with calcium oxalate crystals and, according to the authors, the most probable cause of hyperoxaluria was vitamin C administration, through the conversion of ascorbic acid to oxalate.

### 3.2. Observational Studies

In a retrospective study performed in the USA [30], out of 102 patients admitted to the intensive care service with COVID-19, 73 patients received supplementation with vitamin C and zinc (71.6%; 36 African American, 24 Hispanic, 5 Caucasian). The results of this study showed an overall high mortality and no change in overall survival of patients treated with vitamin C and zinc supplements.

By contrast, one American retrospective study on 79 patients with COVID-19 pneumonia requiring mechanical ventilation found that patients treated with vitamin C had a decreased risk of mortality (OR = 0.39, 95% CI 0.20–0.77) [31].

There are additional observational studies reporting the common use of vitamin C in large cohorts, without providing data regarding outcomes of these patients. A Chinese retrospective study of 596 patients with COVID-19 [32] reported a total of 95 subjects treated with vitamin C, more commonly among patients with cardiovascular disease than without (23.3% vs. 11.8%). However, no data on clinical outcome of the treated group was provided. Another Chinese cross-sectional study of 58 patients with COVID-19 [33] reported the administration of vitamin C in a total of 40 patients, but no information on the outcome associated with this therapy was provided.

### 3.3. Clinical Trials

In one phase-1 clinical trial performed in Iran [34], five patients (M:F = 2:3, mean age 62.8 ± 16 years old) with COVID-19 pneumonia in their final stage (low level of consciousness and/or respiratory distress) were administered a combination of methylene blue-vitamin C (dose: 1500 mg/kg-N-acetyl Cysteine as a compassionate therapy. While four patients improved both their respiratory symptoms and oxygen saturation and were consequently discharged at an average of 10 (4–23) days, one patient discontinued the therapy due to unexpected limitations of the drug preparation and expired on the second day.

A small open-label RCT conducted in Pakistan [35] compared the effect of 1.5 g vitamin C IV every 6 h for 5 days plus lopinavir/ritonavir and oral hydroxychloroquine vs. lopinavir/ritonavir and oral hydroxychloroquine alone in 30 patients per group with severe COVID-19 infection. The authors did not find any difference in the length of ICU stay (*p*  >  0.05), intubation rate (*p*  >  0.05), and mortality rate (three cases in each arm, *p*  >  0.05) between the two groups. On the other hand, the body temperature was lower (36.8 ± 0.5 vs. 37.2 ± 0.7, *p* = 0.001) and SatO_2_ higher (90.5 [88.0–92.0] vs. 88.0 [80.0–91.0], *p*= 0.014) in patients receiving vitamin C as compared to the control group. However, length of hospitalization was higher (8.5 days vs. 6.5 days, *p* = 0.028) in patients managed with vitamin C than in those without.

In a multicenter RCT performed in China [36], 56 patients with severe SARS-CoV-2 infection were randomized to receive either placebo or high-dose IV vitamin C (dose: 12 g/50 mL every 12 H for 7 days, 12 mL/h). No difference was found regarding invasive mechanical ventilation-free days in 28 days and 28-day mortality, while the vitamin C group reported a rise in the PaO_2_/FiO_2_ (229 vs. 151 mmHg, 95% CI 33 to 122) and lower levels of IL-6 on day 7 (19.4 vs. 158.0, 95% CI −301.7 to −29.8). In addition, the ICU mortality of severe patients with SOFA score ≥ 3 was improved after vitamin C administration (HR = 0.22, 95% CI 0.1–0.9).

In an open-label RCT performed in Pakistan [37] on 150 patients with severe COVID-19 infection, the control arm (*n* = 75) received a standard therapy (antipyretics, dexamethasone and prophylactic antibiotics) while the interventional arm (*n* = 75) received the standard therapy in addition to IV vitamin C (dose: 50 mg/kg/day). The latter group became symptom-free earlier (7.1 ± 1.8 vs. 9.6 ± 2.1 days) and reported a shorter duration of hospitalization (8.1 ± 1.8 vs. 10.7 ± 2.2 days) compared to the control group, while no significant difference was found in the need for mechanical ventilation and mortality.

In another open-label multicenter RCT conducted in the USA [38], 214 patients with SARS-CoV-2 infection managed in outpatient clinics, were assigned to receive ascorbic acid (8000 mg), zinc gluconate (50 mg), both agents, or standard of care for ten days. The study was stopped early for futility since conditional power was <30% for the three treatment groups compared with placebo.

The characteristics and key findings of concluded studies, both observational and interventional, are given in Table 1.

### 3.4. Ongoing Trials

Three large RCTs are still ongoing, and data regarding the association between vitamin C administration and patients’ outcomes are still pending. One multi-center, double-blinded, clinical trial [39] performed in the USA on 829 participants, randomized the patients to receive either hydroxychloroquine or ascorbic acid (dose: 500 mg for 3 days, then 250 mg for 11 days orally) as post-exposure prophylaxis for the prevention of SARS-COV2 in adults exposed to the virus. Although the study ended in October 2020, its results have not been published so far. Similarly, in a randomized, single-blinded, two-arm (1:1) parallel-group clinical trial [40] performed in Iran on ICU patients with COVID-19, the treatment group received vitamin A (25,000 IU daily), vitamin D (600,000 IU once), vitamin E (300 IU twice daily), vitamin B and vitamin C (dose: 500 mg, four-time per day) for 7 days, while the control group received neither supplements nor placebo. Since the beginning of this study in April 2020, no results have been published. In addition, a Chinese [41] multicenter prospective randomized placebo-controlled trial has recruited 56 participants with COVID-19 so far, to randomly receive 12 g of IV vitamin C (diluted in 50 mL of sterile water, repeated every 12 h, total dosage 24 g/day) vs. placebo of 50 mL of sterile water, for 7 days. The last update was in October 2020, and results of this study are yet to be published.

## 4. Discussion

This review aimed to summarize current knowledge on vitamin C treatment in patients with COVID-19, based on available publications from last year.

While the theoretical background on vitamin C important role in immune regulation during infections is profound, the therapeutic effect of vitamin C treatment in respiratory infections and critically ill patients is still controversial. The utility of vitamin C treatment in patients with COVID19 is still under investigation worldwide.

As detailed in this review, there are multiple reports of patients with COVID-19 that were treated with vitamin C in different settings, and experienced clinical improvement after the administration of vitamin C administration. However, most publications described in this review are observational, many of them are case reports and small case series, precluding the ability to associate any beneficial effect specifically to vitamin C treatment. These reports should be addressed as clinical observations, as causality could not be claimed between the administration of vitamin C and the improvement of the patients’ medical status. However, some of the preliminary clinical trials have shown encouraging results among the intervention groups using high dose IV vitamin C as mentioned in this review. The results of ongoing RCTs are still pending, and additional high-quality research are required in order to support the potential benefit of vitamin C in the treatment of patients with SARS-CoV-2 infection. Data on a possible effect of vitamin C against SARS-CoV-2 infection in children are to date not available. In addition, there is no evidence to support routine vitamin C supplementation to healthy individuals for the prevention of COVID-19.

Currently, the evidence supporting the therapeutic use of vitamin C in patients with severe COVID-19 is lacking, and no recommendations could be given on this basis. Considering the pathophysiology and theoretical background, together with the preliminary reports and studies aforementioned, the role of vitamin C in the treatment of patients with SARS-CoV-2 infection should be further investigated.

## Figures and Tables

**Table 1:** Characteristics and findings of the concluded observational and interventional studies investigating the effect of vitamin C in patients with a SARS-CoV-2 infection.

First Author, Year and Country	Population	Study Design	Exposure/Intervention with Dosage	Outcomes	Key Findings
Capone S. et al., USA, 2020 [30]	102 patients (median age 63 years) affected by SARS-CoV-2 and managed by intensive care team	Observational, retrospective	Supplementation of vitamin C (plus zinc). Posology not specified	Overall survival	72% received supplementation with vitamin C and zinc. No association between vitamin C and overall survival was observed
Krishnana S. et al., USA, 2020 [31]	152 patients (median age 68 years) affected by SARS-CoV-2, requiring mechanical ventilation	Observational, retrospective	Supplementation of vitamin C. Posology not specified	Overall survival	52% received supplementation with vitamin C. Survival was higher in patients managed with vitamin C (65%, vs. 43%, *p* = 0.007)
Li J. et al., China, 2020 [32]	596 patients (mean age 56 years) affected by SARS-CoV-2, evaluated at the Hospital	Observational, retrospective	Supplementation of vitamin C. Posology not specified	Prognosis of patients with and without cardiovascular diseases	16% received vitamin C. Patients with cardiovascular diseases received vitamin C more frequently (23% vs. 12%, *p* < 0.001) than patients without. No data on prognosis related to the supplementation of vitamin C were available
Liu XH et al., China, 2020 [33]	58 patients (median age 29 years) admitted to the hospital with a SARS-CoV-2 infection	Observational, retrospective	Supplementation of vitamin C. Posology not specified	Clinical characteristics	69% received vitamin C. No data about the association between vitamin C and clinical outcomes were available
Alamdari DH et al., Iran, 2020 [34]	5 patients (mean age 63 years) admitted to ICU for respiratory distress due to SARS-CoV-2 infection	Phase-I clinical trial	Administration of vitamin C (1500 mg/kg) both oral and intravenous	Respiratory symptoms and safety	Four patients showed improvement both in respiratory symptoms and oxygen saturation after vitamin C administration. The patients were discharged in 10 (4–23) days. One patient discontinued the therapy due to limitations of the drug preparation and expired on the second day of admission
JamaliMoghadamSiahkali S. et al., Pakistan, 2020 [35]	A total of 60 patients with a severe SARS-CoV-2 infection: 30 patients (mean age 58 years) received lopinavir/rito- navir and hydroxychloroquine plus vitamin C and 30 (mean age 61 years) only lopinavir/rito- navir and hydroxychloroquine	Randomized open-label clinical trial	Administration of intravenous vitamin C (1.5 g every six hours, total 6 g daily)	The main outcomes were: decrease in mortality, length of hospitalization, and number of patients admitted to ICU. Secondary outcomes were: increase in SpO_2_ and improvements in vital signs and overall wellbeing	Patients managed with and without vitamin C did not differ for any of the outcomes, except for body temperature (36.8 ± 0.5 vs. 37.2 ± 0.7, respectively, *p* = 0.001) and SpO_2_ (90.5 [88.0–92.0] vs. 88.0 [80.0–91.0], respectively *p* = 0.014) on third day of hospitalization. On the contrary, length of hospitalization was higher in patients managed with vitamin C (8.5 days vs. 6.5 days, *p* = 0.028)
Zang J. et al., China, 2021 [36]	56 patients (mean age 67 years) with a SARS-CoV-2 infection admitted to intensive care	Randomized, controlled, clinical trial	Administration of intravenous vitamin C (12 g every 12 h, total 24 g daily) for 7 days	The main outcome was invasive mechanical ventilation-free days in 28 days. Secondary outcomes were 28-day mortality, organ failure severity, and interleukin-6 levels	Patients managed with and without vitamin C showed no difference in terms of invasive mechanical ventilation-free days in 28 days and 28-day mortality. A rise in the PaO_2_/FiO2 (229 vs. 151 mmHg, 95% CI 33 to 122) and lower levels of IL-6 on day 7 (19.42 vs. 158.00, 95% CI −301.72 to −29.79), lower ICU mortality (Hazard Ratio = 0.22, 95% CI 0.1–0.9) in patients with severe multiorgan score failure were observed in patients managed with vitamin C.
Kumari P. et al., Pakistan, 2020 [37]	A total of 150 patients admitted for a SARS-CoV-2 infection: 75 (mean age 52 years) were managed with vitamin C and 75 (mean age 53 years) without	Randomized controlled trial	Administration of intravenous vitamin C (50 mg/kg/day). Length of the intervention not specified	The endpoints were: number of days before symptoms disappearance, length of hospital stay, need for ventilation and mortality	Patients managed with vitamin C were symptom-free earlier (7.1 ± 1.8 vs. 9.6 ± 2.1 days, *p* < 0.0001)) and had a shorter duration of hospitalization (8.1 ± 1.8 vs. 10.7 ± 2.2 days, <0.0001) compared to patients managed without vitamin C. No difference was observed in the need for mechanical ventilation and mortality
Thomas S. et al., USA, 2021 [38]	A total of 214 outpatients with a SARS-CoV-2 infection: 48 (mean age 46 years) were managed with vitamin C, 50 (mean age 42 years) with the standard care, 58 (mean age 44 years) with zinc and 58 (mean age 49 years) with vitamin C and zinc	Randomized factorial open-label trial	Administration of: (1) vitamin C (8000 mg in 2–3 times per day), (2) 50 mg of zinc gluconate, (3) both vitamin C and zinc	The main outcome was the number of days to obtain a 50% reduction in symptoms. Secondary outcomes were: days required to resolve symptoms, cumulative severity score symptoms at day 5, hospitalizations, adjunctive prescribed medications, mortality and safety	The study was ended for lack of benefits after the interim analysis

## Data Availability

Upon reasonable request at the corresponding author.
